# Integrating hospital information systems into foodborne disease surveillance: retrospective assessment, Shenzhen, China, 2024–2025

**DOI:** 10.5365/wpsar.2026.17.2.1394

**Published:** 2026-05-26

**Authors:** Yuchen Xie, Yan Zhou, Zhou Wang

**Affiliations:** a Shenzhen Nanshan District Center for Disease Control and Prevention, Shenzhen, Guangdong, China.; b Shenzhen Field Epidemiology Training Program, Shenzhen, Guangdong, China.; c Shenzhen Center for Disease Control and Prevention, Shenzhen, Guangdong, China.

In China, foodborne diseases require manual reporting with the surveillance network relying on sentinel hospitals as the foundational building blocks of case detection. However, within this framework, traditional manual reporting remains the main bottleneck, often due to heavy clinical workloads. Fu has asserted that automation is the key to solving this problem. (*1*) The transition from passive notification to active early warning requires the leveraging of big data technologies, specifically the automated mining of clinical records to identify potential outbreaks before manual reports are filed. (*1*) In 2024, the Shenzhen Center for Disease Control and Prevention completed the integration of a hospital information system (HIS) into the national surveillance network. A key feature of this digital upgrade is the automated extraction of diagnostic keywords (for example, “acute gastroenteritis” or “food poisoning”) directly from electronic medical records. Operating as a clinical decision support system, this mechanism triggers a pop-up prompt of auto-filled clinical data, enabling real-time detection of suspected cases by requiring clinicians simply to verify and submit the report, rather than manually completing separate reports. This brief report describes an evaluation of the performance of this new digital surveillance system (reporting volume) and user experience (satisfaction) 12 months post-integration.

## Methods

### Study design and data collection

A retrospective assessment of Shenzhen’s digital surveillance system for foodborne disease was performed, using a mixed-methods approach to ensure a comprehensive evaluation.

### System audit (objective data)

De-identified reporting logs were extracted from 53 sentinel hospitals to compare case volumes and reporting timeliness between the manual phase (2024) and the digital integration phase (2025).

#### User survey (subjective data)

A structured questionnaire was distributed to 263 front-line staff. The instrument was developed based on the Beijing Model and adapted for the Shenzhen context. (*2*)

Participants: targeted respondents included clinicians from key departments (for example, paediatrics, infectious diseases and emergency) and public health staff in prevention departments.
Questionnaire structure: the survey consisted of two sections. The first section collected information  about seven demographic characteristics, including age, education and years of experience. The second section was a system evaluation, covering system construction, reporting quality and operational support.
Scoring: most items were rated on a 5-point Likert scale (1 = strongly disagree, 5 = strongly agree) to gather data on whether the respondent thought that the system was working well. Three items recorded the number of dedicated staff and the frequency of system failure and annual training.
An evaluation framework was also included and is described below.

The assessment applied the hierarchical indicator framework established in the Beijing Model, (*2*) which served as the structural basis for evaluating the sentinel hospitals. We applied the original weight coefficients derived from the model's Delphi consultations (authority coefficient ≥ 0.85) to calculate the final scores. Its three primary dimensions are defined here to clarify the metrics used in **Table 1**).

**Table 1:** Evaluation of and satisfaction with the digital system for foodborne disease surveillance (*n* = 263), using the three dimensions of the Beijing Model, (*2*) Shenzhen, China, 2025

Dimension	Subdimension	Indicator (Level 3)	Mean Likert score^a^	Satisfaction rate^b^ or median frequency
**System construction**	**Efficiency**	**Login speed**	**4.59**	**242 (92.0)**
		**Ease of data entry**	**4.57**	**239 (90.9)**
		**Optimization of review process**	**4.61**	**242 (92.0)**
		**Reduction in data entry time**	**4.36**	**229 (87.1)**
	**Data management**	**Convenience of report management**	**4.45**	**234 (89.0)**
		**Convenience of data download**	**4.49**	**237 (90.1)**
	**Analysis**	**Utility of statistical analysis**	**4.38**	**231 (87.8)**
	**Permissions**	**Ease of user permission management**	**4.43**	**234 (89.0)**
	**Assistance**	**Completeness of logic checks**	**4.41**	**231 (87.8)**
	**Compatibility**	**Browser compatibility**	**4.50**	**237 (90.1)**
**Reporting quality**	**Stability**	**Mean frequency of system failures**	**2.03^c^**	**2 times/year^c^**
	**Security**	**Data and permission security**	**4.59**	**237 (90.1)**
	**Timeliness utility**	**Reduction in reporting time**	**4.47**	**234 (89.0)**
		**Utilization for local risk assessment**	**4.30**	**234 (89.0)**
**Operational support**	**Personnel**	**Mean number of dedicated staff**	**4.05^c^**	**3 persons^c^**
	**Knowledge**	**Awareness of case definition**	**4.57**	**239 (90.9)**
		**Awareness of reporting timeliness**	**4.58**	**242 (92.0)**
	**Training**	**Mean frequency of training**	**3.08^c^**	**2 times/year^c^**
		**Satisfaction with training quality**	**4.58**	**242 (92.0)**
	**Hardware**	**Satisfaction with hardware/server**	**4.54**	**239 (90.9)**
	**Credibility**	**Authenticity of entered data**	**4.58**	**242 (92.0)**

^a^ Scores range from 1 (strongly disagree) to 5 (strongly agree) that the system works well.

^b^ Values are *n*(%) unless otherwise indicated.

^c^ These values represent the mean of actual counts, not mean Likert scores.

System construction: focuses on technical efficiency, specifically measuring login speed, ease of data entry and system stability.
Reporting quality: evaluates data integrity, including data security, the timeliness of uploads and the completeness of logic checks.
Operational support: measures “soft” capacity, specifically personnel training frequency and staff knowledge of case definitions.

## Results

The total number of cases of foodborne disease reported in 2024 (pre-integration) was 4329, but after integration of the HIS into the surveillance network in 2025 (post-integration), the number of cases rose to 5403, a 24.8% increase (data not shown). Regarding system efficiency, the survey of 263 front-line staff revealed high satisfaction with the digital workflow. Specifically, 242 out of 263 respondents (92.0%) were satisfied with the login speed (mean score: 4.59/5), and 229/263 (87.1%) indicated that the automation effectively reduced data entry time (**Table 1**).

However, growth was uneven across districts. While Nanshan District recorded a 161% increase in the number of reports (rising from 427 to 1115), Pingshan District saw a 44% decline (dropping from 143 to 80) (data not shown). This disparity aligns with the survey findings across the different evaluation dimensions: while the overall mean score for the System Construction dimension was high (4.48/5), the Operational Support dimension revealed a critical weakness. Specifically, the mean frequency of training during the study period was only 3.08, which corresponds to an actual median frequency of just two training sessions per year (**Table 1**). This highlights a critical gap in personnel readiness.

## Discussion

Our data validate the interoperability dividend, that is, the immediate efficiency gain derived from automated data exchange. The 24.8% spike in case reports signals a structural transformation from passive notification to active capture. By automating the extraction of keywords for symptoms of foodborne diseases, the Shenzhen system has effectively lowered the physical barrier to reporting, supporting the finding that the electronic transfer of data reduces reporting lag. (*3*) Unlike the struggles with manual entry observed in Jiangsu Province where the clinical workload often crowded out public health duties, Shenzhen’s automated approach has successfully embedded surveillance into the workflow of clinicians. (*4*)

Although technology is a force multiplier, it is not the solution to a complex problem. The contrast between Nanshan (a 161% increase in reporting) and Pingshan (a 44% decrease) suggests that surveillance performance is strictly limited by the availability of local resources, such as front-line clinicians to verify data and dedicated public health staff to provide regular training and oversight (for example, the review of the submitted records). (*5*) Alternatively, the decrease in Pingshan might be due to the new system's rigorous automatic rule that removes duplicate reports, which may have successfully filtered out false positives or duplicate entries that were previously over-counted during manual reporting.

This review identified a distinct mismatch between technology and capacity. While the mean score for indicators in System Construction was high (4.48/5), in the Operational Support dimension, mean training frequency was low at 3.08 (reflecting an actual median of two training sessions per year). This is a major weakness in the system. As Feng noted in their study of Ma'anshan City, the primary barrier to effective surveillance is often not the lack of willingness, but the lack of diagnostic clarity among front-line doctors. (*6*) In Shenzhen, while doctors have the tool (the HIS interface), the low training frequency identified by our survey remains a critical bottleneck. In this “human-in-the-loop” system, busy clinicians lacking clear diagnostic criteria may dismiss automated prompts due to alert fatigue (causing underreporting) or hastily confirm them without verifying the prompt to clear the screen (causing misclassification). This mirrors experiences in Europe and United States of America, highlighting that alert fatigue is a universal issue. (*7*) Thus, a system's true sensitivity remains heavily dependent on strict clinical workflow adherence and continuing user training.

Future strategies must pivot from infrastructure investment to prioritizing scenario-based training to bridge the gap between digital tools and front-line competence.

### Limitations

This study has limitations because this retrospective assessment covers only the first year post-integration, and the findings rely on subjective user feedback and may not fully capture long-term trends. Additionally, while variations in reporting volumes between districts were observed, the study’s reliance on purely quantitative data restricts our ability to completely explain these local differences, highlighting the need for future qualitative research.

### Conclusions

Shenzhen’s digital leap has effectively dismantled the reporting volume barrier, demonstrating that interoperability effectively mitigates the burden of manual reporting. However, the stark technology–capacity mismatch, evidenced by the critical lag in training frequency, remains the system's drawback. Future resilience relies on empowering the front-line sentinels rather than merely upgrading the IT infrastructure.
